# The role of microRNAs in ectopic pregnancy: A concise review

**DOI:** 10.1016/j.ncrna.2020.04.002

**Published:** 2020-04-18

**Authors:** Soudeh Ghafouri-Fard, Hamed Shoorei, Mohammad Taheri

**Affiliations:** a Department of Medical Genetics, Shahid Beheshti University of Medical Sciences, Tehran, Iran; b Department of Anatomical Sciences, Faculty of Medicine, Birjand University of Medical Sciences, Birjand, Iran; c Urogenital Stem Cell Research Center, Shahid Beheshti University of Medical Sciences, Tehran, Iran

**Keywords:** miRNA, Ectopic pregnancy, Expression

## Abstract

Ectopic pregnancy (EP) is reported in about 1%–2% of pregnant females and is associated with mortality and morbidity. Several genetic and environmental factors might modulate occurrence of EP. Prediction of EP and patients' follow-up is an important task in management of pregnancy. MicroRNAs (miRNAs) as non-coding RNAs with sizes between 21 and 24 nucleotides have been shown to regulate several aspects of implantation and early fetal life. They have potential role in early detection of EP especially considering their presence in body fluids such as serum. Assessment of their expression in serum might provide a noninvasive method for diagnosis and patients' follow-up. However, data regarding their role in EP is not sufficient due to small sample sizes of the studies. Future studies are required to find miRNAs that regulate expression of EP-associated genes such as *VEGFA*, *EGFR*, *ESR1* and immune response-related genes to provide new diagnostic biomarkers for EP.

## 1 Introduction

Ectopic pregnancy (EP) is defined as occurrence of ovum implantation outside the uterine cavity. The most site of EP is the fallopian tube where it comprises the most frequent source of pregnancy-related death and morbidity in the first trimester. EP is reported in about 1%–2% of pregnant females [1]. About 95% of EP cases happen in the ampullary, infundibular, and isthmic portions of the fallopian tube. The remaining cases happen in the interstitial portion of the fallopian tube, cervix, anterior lower part of the uterus in the scar of a cesarean section, ovary, or peritoneal cavity [2]. The acute symptoms of EP include amenorrhea, irregular light vaginal bleeding and abdominal pain [3]. Although risk factors such as maternal age, partner's cigarette smoking, gravidity, previous spontaneous abortions or EP, tubal pathology and history of infertility have been associated with risk of EP [4], the exact pathogenic events leading to this condition are not clear. A comprehensive analysis of related literature has led to identification of vascular endothelial growth factor A (VEGFA), interleukin-8, interleukin-6, estrogen receptor 1 (ESR1) and epidermal growth factor receptor (EGFR) as the main genes which modulate occurrence of EP [5]. Apart from this group of genes, microRNAs (miRNAs) have been recently regarded as putative participants in the pathophysiology of EP [6]. These non-coding RNAs have sizes between 19 and 24 nucleotides and have been detected in almost all species with high levels of conservation across species [7]. They regulate expression of genes through different mechanisms including pairing with to the complementary sites in the 3′ untranslated region (UTR) of target genes and suppressing their translation [7]. Numerous studies have demonstrated their role in several aspects of the reproductive system physiology, conception, implantation and development of embryo [8]. In the current mini-review, we have summarized their putative function in the pathophysiology of EP.

## 2 Role of miRNAs in implantation

miRNAs are small non-coding RNAs with functional regulatory influences on several genes [9]. They have functional roles both inside the cells and in the extracellular environment where they are delivered via different packaging methods. Such methods of miRNA release modulate intercellular communication in different contexts [10]. A number of miRNAs have been shown to be involved in the process of embryo implantation. For instance, increased expression of miRNA-31 in human endometrium and serum throughout the window of implantation has potentiated this miRNA as a biomarker for optimal receptivity. Such increase in its levels has been parallel with surge in progesterone level and down-regulation of immune-response associated genes such as FOXP3 and CXCL12 [11]. A genome-wide assessment of miRNA signature in cattle has led to identification of miR-26a as a possible peripheral marker for early pregnancy [12]. Assessment of miRNA profile in pregnant, embryonic-mortality, and control cows has revealed differential expression of 27 mature miRNA. Among them, miR-25, -16b, and −3596 could differentiate the pregnancy status at the early phases of pregnancy [13]. miRNA profiling in human endometrial epithelial cells at late proliferative and midsecretory stages of the menstrual cycle has shown up-regulation of 12 miRNAs in the secretory phase. These miRNAs mostly decreased expression of cell cycle genes and inhibit cell proliferation [14]. Others have documented up-regulation of hsa-miR-30b and hsa-miR-30d and down-regulation of hsa-miR-494 and hsa-miR-923 in receptive endometrium compared with prereceptive samples of fertile nonstimulated females [15]. Up-regulation of hsa-miR-30b and hsa-miR-30d has also been reported in receptive endometrium of infertile women [16]. Some studies have pointed to differential expression of miRNAs in placenta during different stages of pregnancy. For instance, miR-371-5p, miR-17-3p, and miR-708-5p were up-regulated in the first trimester placentas compared with the third trimester placentas, whereas miR-125b-5p and miR-139-5p had the opposite trend [17].

## 3 miRNAs and EP

Human studies have sown aberrant expression (down-regulation or up-regulation) of a number of miRNAs in peripheral blood of females with EP. A retrospective assessment of miRNA levels in pregnant females who referred to the emergency department with vaginal bleeding and/or abdominal pain/cramping has shown lower amounts of miR-517a, miR-519d, and miR-525-3p in EP and spontaneous abortion (SA) cases compared with those having viable intrauterine pregnancy (VIP). Yet, expression of miR-323-3p was higher in EP cases, compared with SA and VIP cases [18].

miRNA signature has been also different in embryonic tissues obtained from EPs and controlled abortions. Dominguez et al. have demonstrated downregulation of hsa-mir-196b, hsa-mir-30a, hsa-mir-873, and hsa-mir-337-3p and up-regulation of hsa-mir-1288, hsa-mir-451, and hsa-mir-223 in EP compared to control specimens. In silico analyses have shown interaction of these miRNAs with the mucin type O-glycan production and the ECM-receptor-interaction pathways [19]. Lozoya et al. have assessed expression of Let-7a, miR-132, miR-145 and mir-323-3p in embryonic samples from EP and controlled abortions. They reported decreased amounts of Let-7a and mir-323-3p in ectopic pregnancies, whereas miR-132 and miR-145 amounts were not different between these groups [20].

Lu et al. have assessed expression profile of 21 miRNAs related with pregnancy or placenta in serum of patients with VIP, SA and EP. They reported differential expression of five miRNAs between the mentioned study groups. Notably, they demonstrated lower concentrations of miR-873 and miR-223 in EP compared with the other groups, miR-323 had the opposite trend [21]. Miura et al. have reported differences in expression of a number of cell-free pregnancy-associated miRNAs (miR-323-3p, miR-515-3p, miR-517a, miR-517c, and miR-518b) between EP, SA, or normal pregnancies. However, miR-21 concentrations were different between the three groups [22].

Feng et al. have evaluated expression of miRNAs and core miRNA regulatory modules in Fallopian tube tissues from women with EP. Based on their results, expression amounts of DICER1, let-7i, miR-149, miR-182, and miR-424 and estrogen receptor α could differentiate the tubal implantation region from the non-implantation region. NEDD4, TAF15, and SPEN genes have been recognized as targets of the mentioned miRNAs based on the in silico analyses. Up-regulation of NEDD4 in smooth muscle cell and TAF15 in stromal cell, as well as down-regulation of epithelial cell SPEN were related with occurrence of EP [23].

Zhang et al. have assessed expression of miRNAs in salpingitis-associated EP, EP from other causes and healthy control group. They reported down-regulation hsa-mir-1247 and up-regulation of hsa-mir-1269a in EP cases. The alteration in expression of these miRNAs was even more noticeable in salpingitis-associated EP. Authors suggested aberrant expression of these miRNAs as risk factors for EP [24].

Expression profiling of miRNAs in serum sample of women with EP, spontaneous abortion (SAB) and VIP has shown tens of miRNAs being up-regulated or down-regulated in EP compared with VIP. Their experiments verified the potential of miRNA profiling application in the early first trimester to screen women at risk for EP [25].

**Fig. 1:**
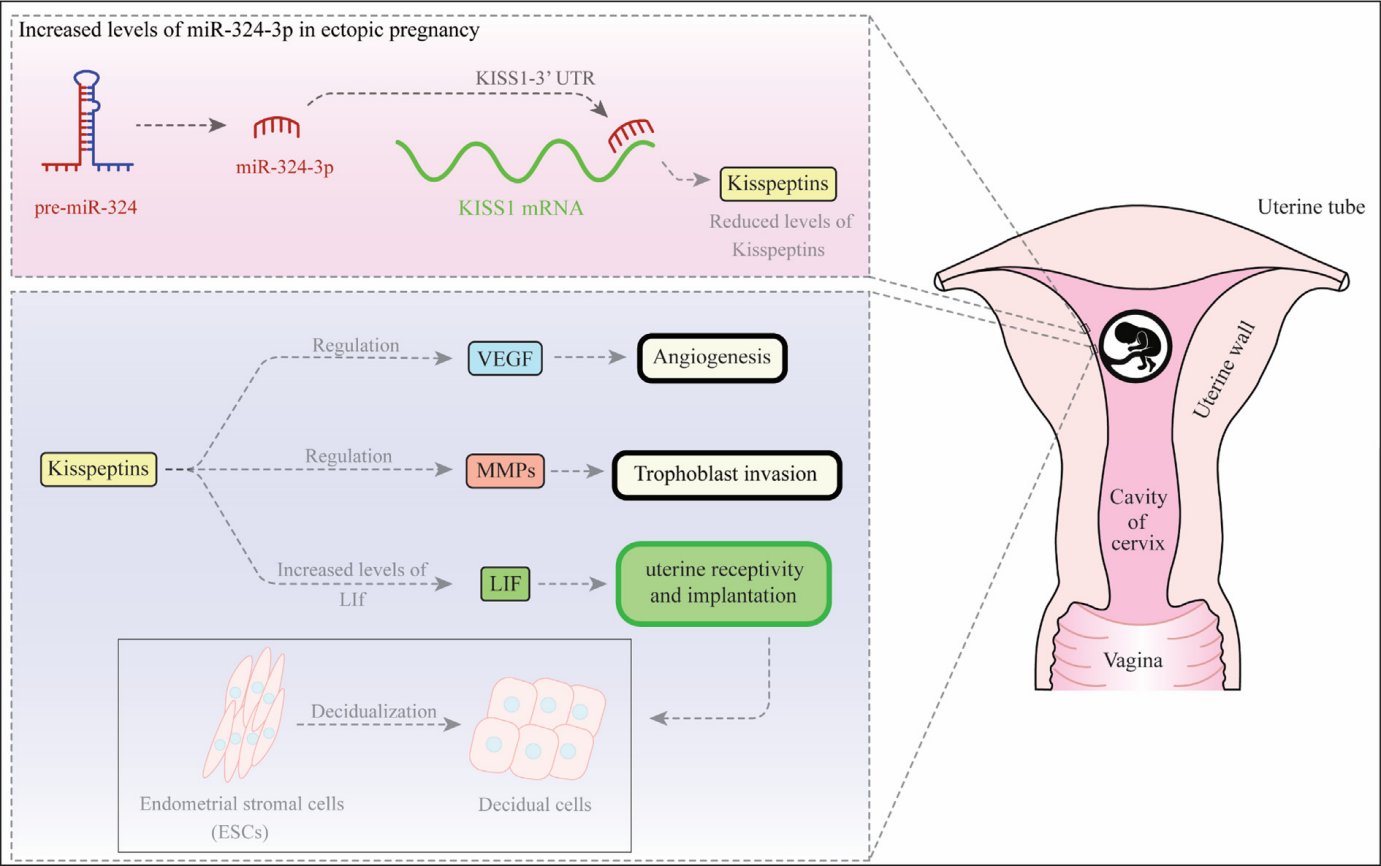
miR-324-3p has been shown to be increased in ectopic pregnancy compared with tissues obtained from voluntary termination of pregnancy. This miRNA has a repressive interaction with 3′-UTR of KISS1 [26]. Kisspeptins have essential roles in implantation through regulation of leukemia inhibitory factor (LIF) [27]. LIF participates to embryo attachment and decidualization. It also regulates expression of MMP and VEGF thus contributing in trophoblast invasion and angiogenesis [28]. Consequently, aberrant expression of miR-324-3p can alter all of these steps.

Fig. 1 depicts the mechanisms of participation of miR-324-3p in the pathogenesis of EP.

Table 1 shows the results of expression assays of miRNAs in EP pregnancies.

**Table 1:** The list of miRNAs whose association with ectopic pregnancy has been assessed (EP: ectopic pregnancy, SA: spontaneous abortion, NP: normal pregnancy).

microRNA	Expression pattern	Numbers of clinical samples	Targets/Regulators	Function	Reference
miR-515-3p, miR-517a, miR-517c	Downregulated	18 EP, 12 SA, 26 NP	–	Cell-free pregnancy-associated microRNAs such as miR-515-3p, miR-517a, and miR-517c could be considered as potential molecular markers of EP.	[22]
hsa-mir-196b, hsa-mir-30a, hsa-mir-873, hsa-mir-337-3p	Downregulated	23 EP, 29 NP	GALNT7, GALNT13, GALNT1, ITGA2, COL1A2	In ectopic and eutopic embryonic tissues, analysis of miRNAs could reveal different expression patterns, which are critical for correct implantation.	[19]
hsa-mir-1288, hsa-mir-451, hsa-mir-223	Upregulated				
miR-517a, miR-519d, miR-525-3p	Downregulated	27 EP, 28 SA, 34 NP	–	miR-323-3p could be considered as an important biomarker for EP.	[18]
miR-323-3p	Upregulated				
miR-132, miR-145	not altered	17 EP, 43 NP	–	In early gestational periods (≤6), the expression levels of miR-132, miR-145, and mir-323-3p did not alter, while Let-7a expression decreases in EP compared to NP. The expression of let-7a could play a key role in the control of normal and EP.	[20]
let-7a	Downregulated				
mir-323-3p	not altered				
let-7i, miR-149, miR-182, miR-424	Upregulated	20 EP, 10 NP	NEDD4, TAF15, SPEN	Mentioned-microRNAs could contribute to the onset of tubal EP through targeting NEDD4, TAF15, and SPEN.	[23]
miR-873	Downregulated	34 EP, 30 SA, 36 NP	–	miR-873 could be considered as a noninvasive biomarker for the detection of early EP.	[21]
hsa-mir-1247	Downregulated	80 EP, 32 NP	–	The abnormal expressions of hsa-mir-1247 and hsa-mir-1269a could be considered as valuable biomarkers for the prediction and treatment of EP.	[24]
hsa-mir-1269a	Upregulated				
miR-324-3p	Upregulated	84 EP, 122 NP	KISS1	Overexpression of miR-324-3p in EP is responsible for decreased expression of KISS1/kisspeptin levels.	[29]
miR-323-3p	Upregulated	30 EP, 30 recurrent miscarriage, 30 NP	–	The high blood level of miR-323-3p could be considered as a valuable biomarker for screening EP.	[30]
hsa-miR-122-3p, hsa-miR-194-3p, hsa-miR-1299, hsa-miR-4755-5p, hsa-miR-378d	Upregulated	5 EP, 5 miscarriage cases, 4 NP	–	Investigating of mentioned-microRNAs in serum samples could be useful as a valuable biomarker for EP.	[25]

## 4 Discussion

miRNAs are secreted from almost all kinds of cells and their amount in the extracellular environment is altered depending on the biological and pathological circumstances [31]. During pregnancy, miRNAs might be released from placenta, embryonic tissues and endometrium. Aberrant implantation of the embryo might change the profile of miRNAs. Consequently, miRNAs have potential role in early detection of EP especially considering their presence in body fluids such as serum. Future studies are needed to assess the clinical implication of miRNAs based on their source of secretion. Attempts to correlate miRNA levels with hCG concentrations failed in some situations [22]. Such finding might imply the presence of another source of rather than placenta for certain miRNAs. Moreover, circulating levels some miRNAs have not been correlated with their tissue expression of the pre-microRNA [29]. So, miRNA profiling in both serum and tissue sample is required for identification of their functions.

Assessment of miRNA expression in serum might provide a noninvasive method for both diagnosis and patients' follow-up. However, data regarding their role in EP is not sufficient due to small sample sizes of the studies. Although a number of studies have verified high sensitivity and specificity values of a single miRNA in detection of EP [22], single miRNAs are not expected to have appropriate sensitivity and specificity values. Yet, combination of miRNA expression signature and biochemical factors is expected to increase these values. Consistent with this speculation, combination of hCG, progesterone, and miR-323-3p has resulted in 77.8% sensitivity (at a fixed specificity of 90%) for detection of EP in a single study [18]. Furthermore, combination of hCG, progesterone and miR-873 had 79.41% sensitivity (at a fixed specificity of 90%) for EP diagnosis [21].

Mechanistic studies in this field are rare. Few miRNA-regulated pathways have been demonstrated to be involved in the pathophysiology of EP among them is LIN28B/Let-7 and KISS1/kisspeptins pathways [20,29]. Future studies are required to find miRNAs that regulate expression of EP-associated genes such as *VEGFA*, *EGFR*, *ESR1* and immune response-related genes. Expression analysis of these miRNAs in tissue samples and sera of patients with EP can provide new insights in the role of miRNAs in the pathogenesis of EP. Such researches would pave the way for introducing miRNAs as biomarkers and therapeutic targets in EP.

Finally, correlation between abnormal miRNA levels and pathological underlying mechanism of EP should be assessed. It is possible that each risk factors for EP influence miRNA signature in a distinct manner.

Taken together, results of preliminary studies have indicated the role of miRNAs in the occurrence of EP and their potential application as biomarkers for this pathological event. Future investigations would confirm whether miRNA signature might predict location of embryo implantation or viability of the pregnancy. These transcripts have potential to be used as diagnostic/prognostic biomarkers for EP. Conduction of high throughput studies can facilitate recognition of this aspect in future.

## Declaration of competing interest

The authors declare they have no conflict of interest.

## References

[1] Barnhart K.T. (2009 Jul 23). Clinical practice. Ectopic pregnancy. N. Engl. J. Med..

[2] Cunningham F., Leveno K., Bloom S. (2010). Chapter 30. The Puerperium. Williams Obstetrics.

[3] Abdulkareem T.A., Eidan S.M. (2017). Ectopic Pregnancy: Diagnosis, Prevention and Management.

[4] Moini A., Hosseini R., Jahangiri N., Shiva M., Akhoond M.R. (2014). Risk factors for ectopic pregnancy: a case–control study. J. Res. Med. Sci.: Off. J. Isfahan Univ. Med. Sci..

[5] Liu J.L., Zhao M. (2016 Feb 1). Prioritization of susceptibility genes for ectopic pregnancy by gene network analysis. Int. J. Mol. Sci..

[6] Kontomanolis E.N., Kalagasidou S., Fasoulakis Z. (2018 Mar 19). MicroRNAs as potential serum biomarkers for early detection of ectopic pregnancy. Cureus.

[7] Bhaskaran M., Mohan M. (2014 Jul). MicroRNAs: history, biogenesis, and their evolving role in animal development and disease. Vet. pathol..

[8] Siristatidis C., Vogiatzi P., Brachnis N., Liassidou A., Iliodromiti Z., Bettocchi S. (2015 Mar-Apr). Review: MicroRNAs in assisted reproduction and their potential role in IVF failure. In Vivo.

[9] Abolghasemi M., Tehrani S.S., Yousefi T., Karimian A., Mahmoodpoor A., Ghamari A. (2020). MicroRNAs in breast cancer: roles, functions, and mechanism of actions. J. Cell. Physiol..

[10] Liang J., Wang S., Wang Z. (2017 Nov 21). Role of microRNAs in embryo implantation. Reprod. Biol. Endocrinol. : RBE (Rev. Bras. Entomol.).

[11] Kresowik J.D., Devor E.J., Van Voorhis B.J., Leslie K.K. (2014 Jul). MicroRNA-31 is significantly elevated in both human endometrium and serum during the window of implantation: a potential biomarker for optimum receptivity. Biol. Reprod..

[12] Ioannidis J., Donadeu F.X. (2016 Mar 3). Circulating miRNA signatures of early pregnancy in cattle. BMC Genom..

[13] Pohler K.G., Green J.A., Moley L.A., Gunewardena S., Hung W.T., Payton R.R. (2017 Aug). Circulating microRNA as candidates for early embryonic viability in cattle. Mol. Reprod. Dev..

[14] Kuokkanen S., Chen B., Ojalvo L., Benard L., Santoro N., Pollard J.W. (2010 Apr). Genomic profiling of microRNAs and messenger RNAs reveals hormonal regulation in microRNA expression in human endometrium. Biol. Reprod..

[15] Altmae S., Martinez-Conejero J.A., Esteban F.J., Ruiz-Alonso M., Stavreus-Evers A., Horcajadas J.A. (2013 Mar). MicroRNAs miR-30b, miR-30d, and miR-494 regulate human endometrial receptivity. Reproductive sciences (Thousand Oaks, Calif).

[16] Sha A.G., Liu J.L., Jiang X.M., Ren J.Z., Ma C.H., Lei W. (2011 Jul). Genome-wide identification of micro-ribonucleic acids associated with human endometrial receptivity in natural and stimulated cycles by deep sequencing. Fertil. Steril..

[17] Gu Y., Sun J., Groome L.J., Wang Y. (2013 Apr 15). Differential miRNA expression profiles between the first and third trimester human placentas. Am. J. Physiol. Endocrinol. Metabol..

[18] Zhao Z., Zhao Q., Warrick J., Lockwood C.M., Woodworth A., Moley K.H. (2012). Circulating microRNA miR-323-3p as a biomarker of ectopic pregnancy. Clin. Chem..

[19] Dominguez F., Moreno-Moya J.M., Lozoya T., Romero A., Martínez S., Monterde M. (2014). Embryonic miRNA profiles of normal and ectopic pregnancies. PloS One.

[20] Lozoya T., Domínguez F., Romero-Ruiz A., Steffani L., Martínez S., Monterde M. (2014). The Lin28/Let-7 system in early human embryonic tissue and ectopic pregnancy. PloS One.

[21] Lu Q., Yan Q., Xu F., Li Y., Zhao W., Wu C. (2017). MicroRNA-873 is a potential serum biomarker for the detection of ectopic pregnancy. Cell. Physiol. Biochem..

[22] Miura K., Higashijima A., Mishima H., Miura S., Kitajima M., Kaneuchi M. (2015). Pregnancy-associated microRNAs in plasma as potential molecular markers of ectopic pregnancy. Fertil. Steril..

[23] Feng Y., Zou S., Weijdegård B., Chen J., Cong Q., Fernandez-Rodriguez J. (2014). The onset of human ectopic pregnancy demonstrates a differential expression of miRNAs and their cognate targets in the Fallopian tube. Int. J. Clin. Exp. Pathol..

[24] Zhang S., Sun Q., Jiang X., Gao F. (2018). Clinical significance of expression of hsa-mir-1247 and hsa-mir-1269a in ectopic pregnancy due to salpingitis. Exp. Therapeut. Med..

[25] Sullivan-Pyke C., Sansone S., Jou J., Koelper N., Stentz N., Takacs P. (2018). Small non-coding RNA as a serum biomarker for non-viable and ectopic pregnancy. Fertil. Steril..

[26] Romero-Ruiz A., Avendano M.S., Dominguez F., Lozoya T., Molina-Abril H., Sangiao-Alvarellos S. (2019 May). Deregulation of miR-324/KISS1/kisspeptin in early ectopic pregnancy: mechanistic findings with clinical and diagnostic implications. Am. J. Obstet. Gynecol..

[27] Cha J., Sun X., Dey S.K. (2012 Dec). Mechanisms of implantation: strategies for successful pregnancy. Nat. Med..

[28] Cao Y., Li Z., Jiang W., Ling Y., Kuang H. (2019 Aug 9). Reproductive functions of kisspeptin/KISS1R systems in the periphery. Reprod. Biol. Endocrinol. : RBE (Rev. Bras. Entomol.).

[29] Romero-Ruiz A., Avendaño M.S., Dominguez F., Lozoya T., Molina-Abril H., Sangiao-Alvarellos S. (2019). Deregulation of miR-324/KISS1/kisspeptin in early ectopic pregnancy: mechanistic findings with clinical and diagnostic implications. Am. J. Obstet. Gynecol..

[30] El-Debakey FE, Zaki KA, Salam MA, Abd-Alla OE, Ibrahim MS. Expression pattern of circulating micro-RNA as A potential diagnostic biomarker for early detection of ectopic pregnancy. Growth.12:13.

[31] Kosaka N., Iguchi H., Ochiya T. (2010 Oct). Circulating microRNA in body fluid: a new potential biomarker for cancer diagnosis and prognosis. Canc. Sci..

